# Gender-based violence (GBV) coordination in humanitarian and public health emergencies: a scoping review

**DOI:** 10.1186/s13031-022-00471-z

**Published:** 2022-06-28

**Authors:** Philomena Raftery, Natasha Howard, Jennifer Palmer, Mazeda Hossain

**Affiliations:** 1 Department of Global Health and Development and Health in Humanitarian Crises Centre, London School of Hygiene and Tropical Medicine, Keppel Street, London, UK; 2 Saw Swee Hock School of Public Health, National University of Singapore and National University Health System, 12 Science Drive 2, Singapore, 117549 Singapore; 3 Centre for Women, Peace and Security, London School of Economics and Political Science, Houghton Street, London, UK

**Keywords:** Gender-based violence (GBV), GBV coordination, Humanitarian emergencies, Public health emergencies

## Abstract

**Background:**

Gender-based violence (GBV) is a global health, human rights, and protection issue, which can increase during emergencies. GBV coordination is an essential component of every humanitarian response, ensuring that, from the earliest phases of a crisis, accessible and safe services are available and prevention and mitigation mechanisms are implemented to reduce GBV. We sought to address the limited evidence on GBV coordination, by reviewing literature on GBV coordination in emergencies, identifying facilitators and barriers influencing effectiveness.

**Methods:**

We conducted a scoping review on GBV coordination in emergencies from 1990 to 2020. Studies explicitly discussing GBV coordination in humanitarian, natural disaster and public health emergencies, in low or middle-income countries, were included. Using thematic analysis, we developed a six-topic framework to synthesise evidence on effective GBV coordination and present recommendations for strengthening GBV coordination in emergencies.

**Findings:**

We included 28 of 964 sources identified, covering 30 different emergency settings across 22 countries. Sources spanned emergency settings, with minimal evidence in public health emergencies and none focussed solely on GBV coordination. Several sources suggested that timely establishment of GBV coordination mechanisms, led by dedicated, experienced coordinators, increased funding and strengthened service provision. GBV risk mitigation was compromised by weak commitment across sectors, poor accountability systems, and limited engagement of affected women. Inclusive GBV coordination, involving national and local actors is vital but engagement efforts have been inadequate and localisation funding targets not yet achieved. Implementation of the GBV Information Management System has reinforced coordination, funding allocation and service provision. While specialist GBV services remain insufficient, emergencies can present opportunities for expansion. Sustainability and long-term impact are compromised by over-reliance on international leadership and funding, weak commitment by governments, and limited attention to GBV prevention.

**Conclusion:**

Despite enhanced global commitments to addressing GBV in recent years, it remains consistently under-prioritised and under-resourced. Recommendations to strengthen GBV coordination in emergencies include: funding dedicated GBV coordination positions across all types of emergencies, building the global GBV coordination workforce, expanding inclusion of national actors and investing in GBV risk mitigation and prevention through multiyear funding. The evidence-based framework for effective GBV coordination presented here, can guide further research in diverse emergencies.

**Supplementary Information:**

The online version contains supplementary material available at 10.1186/s13031-022-00471-z.

## Introduction

### Evolution of gender-based violence (GBV) coordination in emergencies

Gender-based violence (GBV) is a global health, human rights, and protection issue, which often goes underreported and unaddressed [1]. The Inter-Agency Standing Committee (IASC) defines GBV as “an umbrella term for any harmful act that is perpetrated against a person’s will, and that is based on socially ascribed (ie. gender) differences between males and females” p.5 [2]. Humanitarian emergencies, which are becoming increasingly complex and protracted [3, 4], can perpetuate GBV, as vulnerabilities and risks increase and family and community protections are stretched or collapse [5]. During emergencies, coordination between UN agencies, national governments, international, national and local organizations, within the established humanitarian coordination architecture, ensures that responses are effectively delivered [6]. Through collaborative efforts that optimally use available resources and capacities, effective coordination identifies and meets priority needs, addresses gaps and reduces duplication [7, 8]. GBV coordination is defined as an essential component of the humanitarian response, which ensures that from the earliest phases of a crisis, accessible and safe services are available to survivors and that prevention and mitigation mechanisms are put in place to reduce incidents of GBV [7]. Despite expanded international attention and growing evidence on GBV response, risk mitigation and prevention in emergencies, GBV coordination, while recognised as a vital to addressing GBV, is rarely explored systematically or in-depth. This study aimed to fill this gap by synthesising the research evidence on GBV coordination in emergencies, identifying facilitators and barriers to effective coordination.

Deeply entrenched in gender inequality, GBV is often reinforced by patriarchal norms, discriminatory laws, and socio-cultural norms that undermine women’s rights [1, 9]. GBV takes many forms in humanitarian settings, with estimates that one in five refugee or displaced women experience sexual violence [10]. In camp settings for displaced people, intimate partner violence (IPV) is often the most common reported form of GBV [11–13]. Public health emergencies such as epidemics and pandemics also increase GBV-related risks and limit access of survivors to services, due to lockdowns and staff and resource constraints [14, 15].

GBV coordination in humanitarian emergencies falls within the protection cluster in the United Nations (UN)-led international humanitarian cluster system adopted in 2005 [16], with the GBV Area of Responsibility (AoR), led by UNFPA, acting as the global forum for GBV coordination since 2006 [7]. The GBV AoR leads GBV coordination in non-refugee emergencies [8], while in refugee contexts, UNHCR takes the lead under the refugee coordination model, often in collaboration with UNFPA [7, 17, 18]. At the country-level, GBV coordination ensures a multi-sectorial and multi-level response for survivors, including Health, Mental Health and Psychosocial Support (MHPSS), Legal aid, and Livelihoods [7]. The Gender-Based Violence Information Management System (GBVIMS) enables GBV service providers to safely and ethically collect, store, analyse, and share, data related to reported GBV incidents which informs coordination and programming [19]. National and field-level coordination mechanisms often have different, but complementary functions [7].

Addressing GBV requires a broad multi-sectorial, interagency approach, therefore, successful GBV coordination, depends on a wide variety of actors collaborating to achieve safe, ethical and comprehensive GBV programming [7]. GBV coordination promotes a shared understanding of GBV amongst humanitarian, national and local actors, ensuring that GBV minimum standards and guiding principles are known and that GBV is prioritized by response leadership, donors and actors [7]. Crucially, the 2015 guidelines state that all humanitarian actors must act under the assumption that GBV is occurring, regardless of the existence of evidence and outline responsibilities and actions to be taken by each sector to identify and mitigate GBV risks [2]. The GBV sector is closely linked with the work of the larger Protection sector, and also with the other areas of responsibility within the Protection sector, particularly Child Protection [7, 16]. Close coordination with the Health sector, is required for implementation of the Minimal Initial Service Package (MISP), which provides guidance on sexual and reproductive health and GBV service provision in emergencies, and Clinical Management of Rape (CMR) services [7, 20]. MHPSS responsibilities are usually attached to the Health or Protection clusters or addressed within a cross-sectoral working group [7]. Other sectors with specific GBV risk mitigation responsibilities include Water, sanitation and hygiene (WASH), Shelter, Education and Livelihoods. Systemic gender inequality is recognized as a root cause of GBV, therefore, gender equality programming is critical and protection against sexual exploitation and abuse (PSEA) is also often closely linked to GBV coordination [7, 16]. The cross-cutting nature of GBV programming can make coordination of diverse actors operating within complex, emergency contexts challenging, which can compromise GBV survivors’ access to services [13].

### GBV policy context advances

International attention and commitment to addressing GBV in emergencies has rapidly expanded in recent years [21]. The UN Security Council has adopted seven ground-breaking resolutions which frame the Women, Peace, and Security agenda over the past two decades [22, 23]. GBV funding flows and accountability mechanisms increased considerably following the 2013 multi-stakeholder global Call to Action on protection from GBV in emergencies [1] which mobilized attention and high-level commitment from global actors and donors [21]. Call to Action partners launched 5 year road maps in 2015 and 2021 [5, 24] with outcome two, focused on the humanitarian architecture, promoting effective and accountable inter-agency GBV leadership and coordination [24]. Several international best practice standards, guidelines, training resources and technical tools to support GBV coordination in emergencies have also been developed [21], including the 2015 guidelines for integrating GBV interventions in humanitarian action [25]; the 2010 handbook (updated in 2019) for coordinating GBV interventions in emergencies [7, 16]; a 2014 set of core competencies considered necessary for effective GBV prevention and response programming and inter-agency coordination [26]; and a 2020 set of minimum standards for GBV programming in emergencies [27]. At the operational level, in 2014 creation of GBV AoR Regional Emergency GBV Advisor (REGA) roles for deployment in Level three emergencies, was a successful follow-up to these global-level investments. GBV is a core component of UNHCR’s protection mandate and GBV and gender equality are priority areas in UN Humanitarian Coordinators’ Terms of Reference.

Within this rapidly evolving context, understanding what influences effective GBV coordination in different contexts is critical. While several systematic reviews address GBV prevention and response in humanitarian settings, literature exploring GBV coordination is limited. This review aimed to examine literature on GBV coordination in humanitarian and public health emergencies, to identify facilitators and barriers to effective GBV coordination, and to draw out lessons for strengthening GBV coordination in emergencies.

## Methods

### Study design

We conducted a scoping review from October 2020 to January 2021 using Arksey & O’Malley’s five-stage approach [28, 29]. The term “emergencies” refers to situations of armed conflict or natural disaster, involving population displacement, or public health emergencies such as outbreaks, epidemics or pandemics [30].

### Research question

Our research question was intentionally broad to capture a range of sources [28]: ‘What is the existing evidence on GBV coordination in emergencies, including facilitators and barriers influencing its effectiveness?’.

### Identifying potential sources

We initially searched six databases (i.e. Web of Science, Scopus, Pubmed, Medline, EMBASE, Global Health systematically using search terms related to three concepts: (a) humanitarian response/crisis/emergency; (b) emergency response coordination; and (c) gender-based violence/GBV (Additional file 1: Box 1 Search strategy). Secondly, we searched 12 relevant websites purposively (i.e. GBV AoR, OCHA, UNHCR, UNFPA, UN Women, UNICEF, WHO, International Rescue Committee [IRC], CARE, Women’s Refugee Council, ALNAP, Interagency working group on reproductive health in crises (IAWG)) using ‘GBV coordination’ and related search terms. Finally, we searched reference lists for additional relevant sources.

### Selecting sources

We screened potential sources by title and abstract and the remainder by full text against eligibility criteria (Table 1). We included humanitarian, natural disaster and public health emergency settings in low or middle-income countries including emergency onset, relief and recovery phases. We included any affected populations (e.g., refugees, service providers, emergency responders, policy professionals), any study design (e.g., qualitative, quantitative, evaluation), published from 1990 to 2021 in English. We only included sources explicitly discussing GBV coordination and excluded those reporting interventions or approaches to GBV prevention and response that did not explicitly discuss GBV coordination. Conference abstracts, training materials, social media, media, and guidance and policy documents were excluded.

**Table 1:** Eligibility criteria

Category	Inclusion criteria	Exclusion criteria
Context	Humanitarian and public health emergency settings including relief, and recovery phases	Other settings
		Pre-emergency, non-emergency settings e.g. preparedness
Topic	Studies explicitly mentioning GBV coordination and containing information pertaining to at least one of the key topics of GBV coordination identified as: Implementing a GBV sub-cluster; prioritisation, advocacy and access to resources; GBV risk mitigation and integration; localisation; data and information management; coordination for service delivery.	Studies that did not explicitly mention GBV coordination
		Reviews or evaluations of individual GBV response or prevention interventions or approaches
		Other topics
Source type	Research articles	Conference abstracts covering material in a publication
	Systematic/scoping reviews	Training materials
	Technical reports with a research component	Individual/household case studies
	Organisational reports	Protocols, methods description only
	Evaluations	Social media/media, audio/video
		Guidance and policy documents
Study design	All study designs	No research component/entirely theoretical
Participants/population	Staff of UN, international and national organisations working on GBV in emergency settings, GBV service providers and affected populations in emergency and humanitarian settings	Populations in non-emergency/non- humanitarian settings
Publication year	1990—January 2021	Pre-1990
Language	English	Other languages with no English abstract

### Charting and synthesis

We kept the definition and scope of GBV coordination broad to capture a range of data. We extracted data using the six elements outlined in the Call to Action roadmap outcome two, i.e. GBV Sector coordination; coordination between GBV, PSEA, and gender equality; coordination on risk mitigation; integration of GBV; localization; resources and advocacy [24]. We synthesised data on each topic then used an iterative approach, informed by Ritchie & Spencer’s framework method [31], and the coordination functions outlined in the GBV coordination handbook [7], to develop six synthesised themes: (1) Implementing a GBV sub-cluster; (2) GBV prioritisation, advocacy and access to resources; (3) GBV risk mitigation and integration; (4) GBV localisation; (5) GBV data and information management; and (6) GBV coordination to support service delivery. We used thematic analysis to identify and summarise data on facilitators and barriers to effective GBV coordination within each theme.

## Results

### Source characteristics

**Fig. 1:**
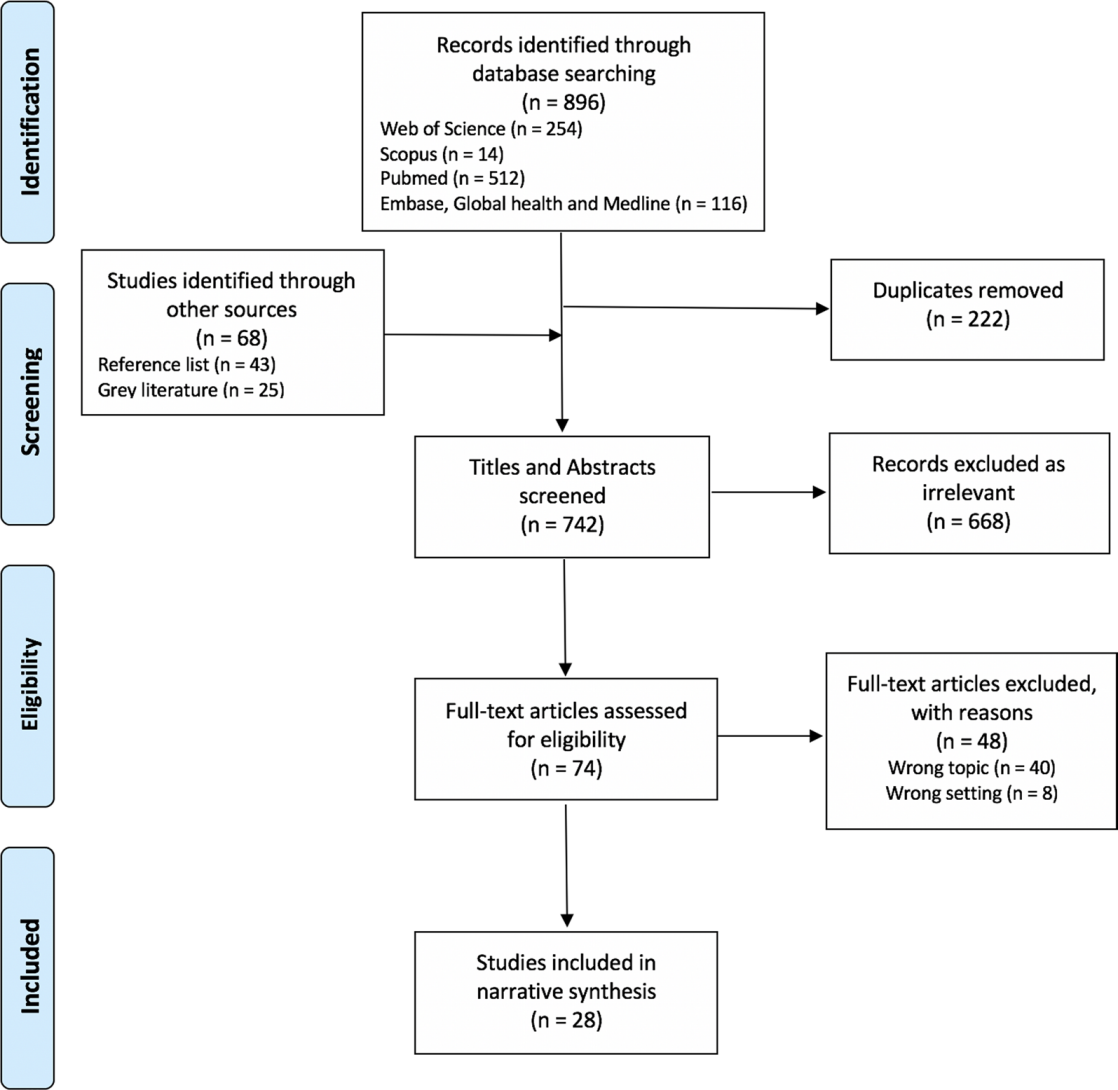
PRISMA flow diagram

We included 28 sources of 964 identified (i.e. 896 from databases, 25 from websites, 43 from reference lists). Figure 1 presents the PRISMA flow diagram and Table 2 provides characteristics of included sources. All studies were conducted and published between 2008 and 2020. Most [23] included frontline ‘field-level’ perspectives covering 30 different emergency settings across 22 countries. (i.e. 12 Middle East and North Africa region—primarily the Syrian refugee crisis (10/12), 12 sub-Saharan Africa, six Asia–Pacific region, four Americas, 10 multi-country, 5 global-level), although none focussed solely on GBV coordination. Three sources discussed public health emergencies (i.e. 2013–16 West Africa Ebola epidemic, 2012 cholera outbreak in Haiti, COVID-19 within the Syrian refugee crisis), five covered natural disasters (i.e. Pakistan floods, Ethiopia drought, Indonesia tsunami, Nepal earthquake), and the remainder involved humanitarian settings at various stages of crisis. Less than half [11] were peer-reviewed articles and 17 were organisation reports, evaluations or non-peer reviewed research, three of which were independent evaluations.

**Table 2:** Source characteristics, including: author, year published, study design, population, country context and GBV coordination topic covered for each source

References	Study design	Population	Country	Emergency context	Framework for effective GBV coordination theme covered					
					Implementing a GBV sub-cluster	Prioritisation, advocacy and access to resources	GBV risk mitigation, and integration	GBV localisation	Data and information management	Coordination to support service delivery
Amiri [32]	Systematic literature review	Syrian refugees Women & Girls	Jordan	Syrian refugee crisis	x					x
Chynoweth [37]	Original research, qualitative	Iraqi refugeesWomen & Girls	Jordan	Iraqi refugee crisis	x					x
Davoren [52]	Original research, qualitative	Women & Girls	Haiti	Post-earthquake IDP setting & cholera outbreak	x	x	x			x
GBV AoR Localization Task Team [50]	Report using mixed-methods approach, employing both qualitative and quantitativemethods	Refugees and IDPs Women & Girls	Iraq Nigeria South Sudan, Whole of Syria Turkey Hub	Internal & Syrian crisis migrant, refugee, IDPInternal Conflict & IDP Internal Conflict & IDPs Syrian crisis IDP remote	x	x		x		x
Krause [33]	Original research, qualitative	Syrian refugee Women & Girls	Jordan	Syrian refugee crisis	x		x			x
Hanley [41]	Evaluation using mixed quantitative methods	Refugees Women & Girls	Lebanon	Syrian refugee crisis	x	x	x	x	x	x
Hanley [19]	A synthesis of key findings from evaluations of UNHCR approaches to GBV in humanitarian crises 2016–18	Refugees Women & Girls	Global		x	x	x	x	x	x
Henttonen [54]	Original research, mixed qualitative and quantitative methods	Refugees/GBV survivors/Female Adult	Northern Uganda	2006 Internal Conflict & IDPs	x	x	x			x
Horn, [11]	Original research, qualitative	Displaced populations/Female Adult	Kenya	Kakuma refugee camp	x		x			x
International Rescue Committee [44]	Rapid assessment report using qualitative methods	Syrian refugee Women & Girls	Lebanon	Syrian refugee crisis	x					x
International Rescue Committee [42]	Discussion paper using document review	IDPs, Refugees Women & Girls	Haiti Pakistan Kenya and Democratic Republic of Congo (DRC)	2010 post-earthquake 2010 Floods 2011 Somali Refugees fleeing famine in Dadaab refugee camp 2012 Internal Conflict & IDPs	x	x	x			x
International Rescue Committee [43]	Discussion paper using document review	IDP, Refugees and national population Women & Girls	Central African Republic (CAR) South Sudan Iraq Sierra Leone	2013 Internal Conflict & IDPs 2013–15 Internal Conflict & IDPs 2014 Islamic state Conflict & IDPs 2013–16 Ebola outbreak	x	x	x	x		x
International Rescue Committee [21]	Report using desk review and key informant interviews	Refugees and IDPs Women & Girls	Global		x	x		x		x
International Rescue Committee [51]	Report using mixed qualitative and quantitative methods	Refugees and IDPs Women & Girls	Global			x		x		
International solutions group [47]	Independent evaluation commissioned by UNFPA using qualitative methods	Global level and country level International and national organisations responding to emergencies	Global and country level Kenya DRC and Colombia	Dadaab refugee camp Internal Conflict & IDPs Internal Conflict & IDPs	x	x			x	x
Irish Consortium on GBV [53]	Report using qualitative methods	Syrian Refugees Women & Girls	Lebanon	Syrian refugee crisis	x	x		x		x
Landegger [39]	Original research, qualitative	Displaced populations/Female Adult	Northern Uganda	Internal Conflict & IDPs	x	x	x		x	x
Myers [34]	Original research, qualitative	Post-earthquake IDP setting Women & Girls	Nepal	Post-earthquake IDPs	x		x			x
Onyango [45]	Review of five assessments	Humanitarian settings Refugee and IDP Women & Girls	Pakistan Chad Indonesia Kenya and Haiti	2003 Afghan refuges 2004 Sudanese refugees from Darfur 2005 Tsunami IDPs 2008 Post-election Violence IDPs 2011 Earthquake	x		x			x
Robbers [59]	Systematic literature review	Refugees Women & Girls	Global		x		x			x
Rothkegel [48]	Evaluation using primarily qualitative methods	Refugees Women & Girls	Tanzania DRC Yemen Nepal and Georgia	Refugees Returnees Urban populations Bhutan refugees Chechen and Kits refugees & IDPs	x	x	x	x	x	x
Steets [40]	Independent evaluation using qualitative methods	Refugees and IDPs	Northern Uganda	Internal Conflict & IDPs	x		x	x		x
UNFPA, UNHCR, IRC, UNICEF, IMC[58]	Evaluation using qualitative methods	Syrian Refugees Women & Girls	Jordan Lebanon Turkey Iraq	Syrian refugee crisis	x		x			
UNFPA [15]	Evaluation using qualitative methods	Syrian Refugees Women & Girls	Cross border operations into Syria from Turkey Jordan Lebanon and Iraq	Syrian refugee crisis	x	x	x	x	x	x
UNICEF [38]	Evaluation using qualitative and quantitative methods	Refugees and IDPs Women & Girls	CAR Jordan Lebanon Nepal Pakistan Somalia South Sudan DRC	Internal Conflict & IDPs Syrian refugee crisis Syrian refugee crisis Post-earthquake Floods/earthquake Internal Conflict & IDPs Internal Conflict & IDPs Internal Conflict & IDPs	x	x	x	x	x	x
Wayte [36]	Original research, qualitative	Conflict IDP setting Women & Girls	Timor-Leste	Internal Conflict & IDPs	x					x
Womens Refugee Council [46]	Evaluation using qualitative methods	Refugees and IDPs Women & Girls	Lebanon Tanzania Ethiopia	Syrian refugee crisis Burundi refugees Drought	x	x	x	x	x	x
Womens Refugee Council [55]	Report using document review	Refugees and IDPs Women & Girls	Global		x	x	x	x		x

### Overview of GBV coordination

**Fig. 2:**
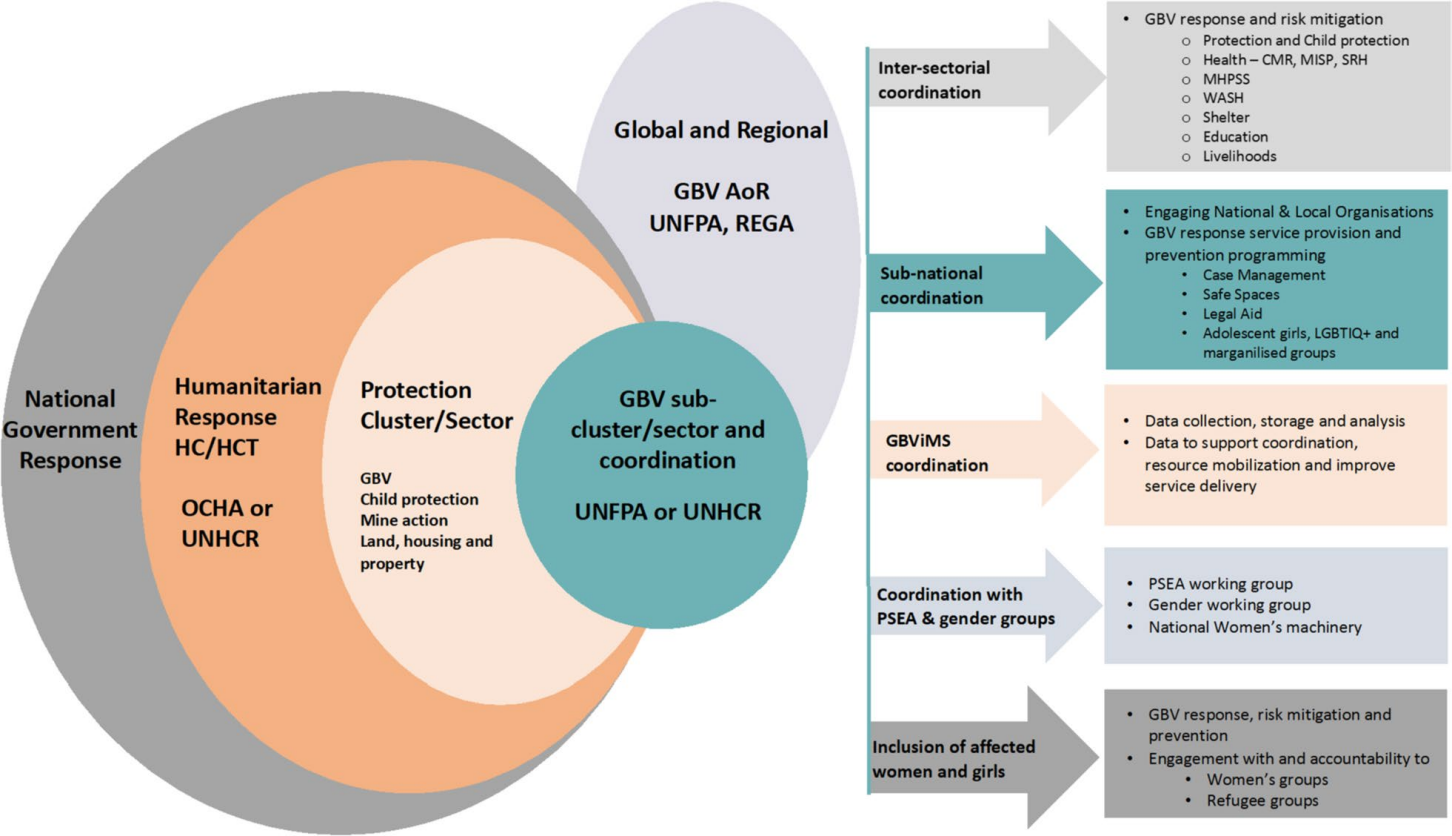
Graphic overview of GBV coordination from global to frontline level. GBV AoR = GBV Area of Responsibility; UNFPA = United Nations Population Fund; REGA = Regional Emergency Gender Based Violence Advisor; UNHCR = United Nations High Commissioner for Refugees; OCHA = Office for Coordination of Humanitarian Affairs; HC/HCT = Humanitarian coordinator/Humanitarian country team; PSEA = Prevention of sexual exploitation and abuse; CMR = Clinical management of rape; MISP = Minimum initial service package; SRH = Sexual and reproductive health; LGBTIQ +  = Lesbian, Gay, Bisexual, Trans and gender diverse, Intersex, Queer and questioning

To visualise the complex matrix of agencies, relationships, and mechanisms constituting GBV coordination, we developed a graphic overview of GBV coordination from global to frontline level (Fig. 2) based on descriptions of GBV coordination in the GBV coordination handbook and guidance documents [7, 16].

### Thematic analysis

**Fig. 3:**
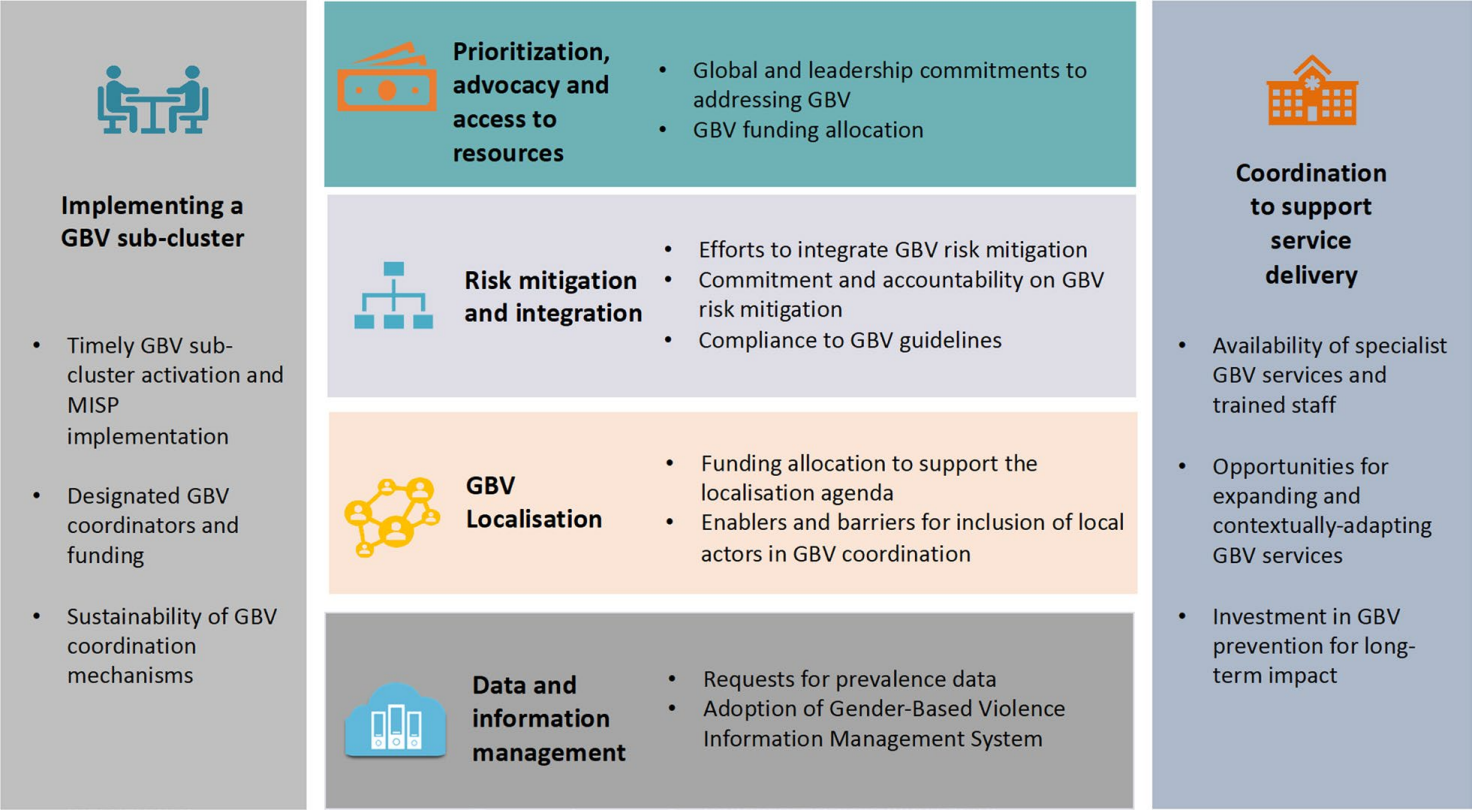
Evidence-based framework of themes influencing effective GBV coordination

Table 3 presents facilitators and barriers to effective GBV coordination identified through the scoping review, Fig. 3 presents an evidence-based framework of themes influencing effective GBV coordination, and evidence supporting each theme is summarised below.

**Table 3:** Facilitators and barriers to effective GBV coordination identified through the scoping review

GBv coordination framework theme	Facilitators	Barriers
Implementing a GBV sub-cluster	1. Timely GBV sub-cluster activation and MISP implementation	1. Late or non-activation of a GBV coordination mechanism and MISP implementation
	2. Designated interagency GBV coordinators and funding	2. Late or short-term deployment of coordinators
		3. Limited government engagement compromised sustainability
Prioritisation, advocacy and access to resources	3. Increased high-level commitments to combatting GBV	4. Insufficient and inconsistent GBV funding allocation
Risk mitigation and integration	4. Roll-out of GBV guidelines enhanced efforts to integrate GBV risk mitigation	5. Low commitment and accountability on GBV risk mitigation across sectors
		6. Non-compliance to GBV guidelines exacerbates GBV risks
Localization	5. Long-term capacity building, mentoring and partnerships with UN agencies and INGOs and mentoring of local and national NGOs	7. Minimal progress on funding allocation to support the localisation agenda
		8. Lack of global good practice standards to guide localization efforts
		9. Exploitative and unequal partnerships
		10. Language and cultural barriers to local and national NGOs engaging in coordination mechanisms
Data and information management	6. Adoption of Gender-Based Violence Information Management System	11. Donors requests for GBV prevalence data delay funding hampering implementation
Coordination to support service delivery	7. Emergencies present opportunities for expanding and contextually-adapting GBV services	12. Insufficient specialist GBV services and trained staff
		13. Limited investment in GBV prevention programming for long-term impact

#### Implementing a GBV sub-cluster

All 28 sources included reflections on implementing a GBV sub-cluster. Timely GBV sub-cluster activation, with dedicated GBV coordinators and funding were noted as critical for effective GBV coordination. However, over-reliance on international funding, technical support and leadership, compromised sustainability.

##### Timely and clear GBV sub-cluster activation and MISP implementation

Several sources noted a growing awareness and commitment to addressing GBV early in humanitarian response [32–37]. UNICEF in 2016, reported implementing rapid GBV responses following declarations of Level 3 emergencies in Lebanon, Jordan, South Sudan, and Nepal [38]. In Nepal, strong MISP coordination was driven by committed leadership from the Ministry of Health, leveraging existing relationships between government, international non-governmental organization’s (INGOs), UN agencies, and national actors, resulting in the rapid establishment of GBV coordination and a GBV referral pathway [34]. Uganda’s protracted humanitarian emergency was one of the first pilots of the Humanitarian Cluster Approach in 2006 and was praised by humanitarian actors and GBV specialists for improving GBV coordination [39]. The Uganda GBV sub-cluster reduced duplication, enhanced GBV services quality, defined a standardized referral pathway, developed a system for collecting GBV data, and formed a consortium to implement common trainings and mobilise funds [39, 40]. The establishment of sub-national or decentralised coordination structures which complement national level functions was also deemed beneficial in several settings. In Lebanon and Northern Uganda, for example, UNHCR and UNFPA had decentralised coordination and delegated authority to field offices, allowing them to better adapt the response to the local context and engage more operational local actors [39, 41].

Conversely, late or non-activation of a GBV coordination mechanism and MISP implementation compromised GBV service delivery. For example, the GBV sub-cluster in Pakistan’s 2010 emergency, was established almost two months after emergency declaration [42], in 2014 in Iraq was activated seven months after emergency onset, and not at all in Sierra Leone during the 2013–16 Ebola epidemic [43]. With no GBV coordination mechanism in place in Sierra Leone, and limited funding, GBV actors were unable to address gaps left by the overwhelmed health sector, leaving GBV survivors without critical services [43], pointing to an important gap in GBV coordination in public health emergencies. Minimal coordination and lack of adherence to international standards in 2011, hindered refugee access to appropriate GBV services at the start of the Syrian refugee crisis, in Lebanon [44]. Weak MISP coordination in Jordan in 2007, impacted GBV response, although improvements were noted during the Syrian refugee response [33, 37, 45]. Nevertheless, coordination in urban settings was weaker than camp coordination, because dispersed refugees were less visible [32, 33]. In Timor-Leste 2006 response, an interagency MISP coordinator was not appointed, with GBV response leadership and advocacy consequently lacking [36], and in Indonesia lack of coordination hindered MISP implementation [45]. Complex UN coordination systems in Iraq in 2014, with the cluster system activated for internally displaced persons (IDPs) in parallel to the UNHCR-led system for refugees, complicated GBV response work, leading some to criticise GBV coordination as ‘confusing’ or ‘inefficient’ [43].

##### Designated GBV coordinators and funding

Strong coordinators with good leadership qualities and dedicated time and funding for coordination were highlighted as critical facilitators for effective GBV coordination. In Lebanon, national coordination was considered strong, attributed to having dedicated coordinators with allocated budgets [41, 46]. In Northern Uganda, introduction of resourced GBV coordinators at national, regional, and district levels, chosen for their “good leadership qualities”, was also deemed a coordination facilitator [39]. In the Syria response, strong GBV coordination and contextual understanding, facilitated effective use of limited resources and improved trust and accountability among stakeholders, improving GBV service delivery [15]. In 2016, UNICEF noted that successful implementation of GBV programming in multiple settings was enabled by deployment of GBV specialists for an extended period [38], and in South Sudan, deployment of GBV AoR regional emergency GBV advisors (REGAs), to inform development of the 2015 humanitarian response plan, increased funding allocated to GBV [43]. Furthermore, in settings where GVBIMS had been most successful, strong and committed coordinators had facilitated analysis and reflection on data [47]. However, recurrent challenges were noted across responses related to human resources, including GBV expertise often being deployed late in a response and rapid staff turnover, creating gaps and inconsistencies [19, 38, 42, 43, 46, 48, 49]. Additionally, sources indicated that GBV coordinators were often junior, short-term and expected to fulfil roles beyond coordination, diluting their focus [4, 41, 50]. Moreover, lack of GBV expertise in senior management positions prevented prioritization of GBV within humanitarian response plans [51].

##### Sustainability of GBV coordination

In many settings, GBV coordination relied on international donor funding, technical support and leadership, with limited ownership of government, compromising sustainability. In Northern Uganda, for example, concerns were raised about government ownership of GBV activities, political commitment to GBV, and capacity to sustain GBV coordination and service delivery [39]. In the Haitian post-earthquake and cholera epidemic emergencies, the GBV sub-cluster was criticised for not working more effectively with the Women’s Ministry in the early stages [52]. GBV programming through the humanitarian response plan in Lebanon created a parallel system and there was a need to strengthen support for government leadership in GBV coordination [53], and both GBV coordination and services remained reliant on international funding [41, 46].

#### Prioritisation, advocacy and access to resources

Seventeen sources noted prioritisation, advocacy and access to resources as critical for GBV coordination at global and frontline levels [15, 19, 21, 38, 39, 41–43, 46–48, 50–55]. While in recent years, high-level commitments to addressing GBV in emergencies have encouraged investments, GBV is still not systematically prioritised and funding remains insufficient and inconsistent across settings.

##### Increased commitments to addressing GBV

Since 2013, the Call to Action has promoted senior leaders in donor and implementing agencies to prioritise GBV, galvanising collective action, accountability, and investments [21]. Following the World Humanitarian Summit in 2016, initiatives such as the *Grand Bargain* and *New Way of Working* focused on increasing multi-year, collaborative, and flexible funding and planning, moving towards longer-term GBV strategies [56, 57]. A 2017 global report on the impact of the Call to Action noted that non-governmental organization’s (NGOs) were increasingly accessing multi-year GBV funding and donors were playing a role in ensuring that funding was channelled through the country response plans and that GBV is addressed within funding proposals [21]. Support from senior leadership within UNHCR and UNICEF was reported as a key factor in prioritising GBV in Lebanon, Jordan, Somalia, and South Sudan, demonstrating the importance of leadership and advocacy in harnessing resources [38, 41, 46]. A multi-country source noted that implementation of the Call to Action Road Map strengthened humanitarian GBV responses in Lebanon and Ethiopia [46].

Despite this progress, humanitarian leadership and sectoral actors on the ground, overwhelmed with competing priorities, often dismiss GBV as non-essential, particularly in acute emergency stages [21, 42]. Lack of GBV technical capacity in country, particularly at the onset of an emergency, when funding priorities are being agreed, can mean GBV is not prioritised, and limits investment in GBV throughout the emergency [38]. Donors can accentuate this by not allocating specific funding for GBV as a life-saving intervention [51]. Sources described significant delays by humanitarian leadership in Iraq and Sierra Leone in including GBV analysis in emergency reporting [43]. In Haiti’s 2010 earthquake and cholera emergency responses, weak coordination was blamed for GBV not being included among high-level priorities, and inadequate adherence to international standards by humanitarian responders [52].

##### Insufficient and inconsistent GBV funding allocation

GBV funding is insufficient and inconsistent across settings and is often subsumed within protection sector budgets, making it difficult to track specific GBV investments [19, 51]. One study reported that according to the office for humanitarian affairs (OCHA) financial tracking service, between 2016 and 2018, GBV only accounted for 0.12% of all humanitarian funding [51]. Another noted that donors and common funding pools did not consistently fund GBV during emergencies [43]. For example, 2014 humanitarian response plans for Central African Republic, South Sudan, and Iraq, only fulfilled 5.2%, 20.9%, and 5.5% of GBV funding requests, respectively [43]. In the 2015 South Sudan response in Ethiopia, only 2% of the budget was allocated to GBV [19] and out of $1.4 billion funding requested following the 2010 Haiti earthquake, only $5 million (0.3%) was allocated for GBV programs [42]. Conversely, in Lebanon, OCHA prioritized GBV in its call for proposals, resulting in overall funding for the sector reaching 38%, though this remained insufficient compared with the needs [19, 41, 46]. In Dadaab refugee camp in 2011, one year after famine declaration, reported cases of GBV increased by a third, while GBV funding was cut in half [43]. Although UNFPA reported being able to scale to meet growing needs in the Syrian crisis response, through successful advocacy for non-earmarked predictable, multi-year funding, they struggled to increase budgets to meet expanding GBV needs due to COVID-19 [15].

#### Risk mitigation and integration

Nineteen sources highlighted the importance of GBV risk mitigation and cross-sector coordination in emergency responses [11, 15, 19, 33, 34, 38–43, 45, 46, 48, 52, 54, 55, 58, 59]. Efforts to integrate GBV risk mitigation in humanitarian settings are compromised by weak commitment and accountability across sectors, and this non-compliance exacerbates GBV risks for women and girls.

##### Enhanced efforts to integrate GBV risk mitigation

Since 2016, when UNICEF noted a lack of systematic integration of GBV risk mitigation by clusters/sectors [38], the roll-out of the GBV guidelines has played a critical role in increasing commitments to GBV risk mitigation, with several good practices emerging. By establishing a focal points network in Jordan and in Lebanon, UNHCR managed to integrate GBV risk mitigation into other sectors [19, 41]. GBV coordination in Lebanon used a mentorship approach to introduce the GBV guidelines to five priority sectors as part of the roll-out of the guidelines in 2017 [46], and facilitated intersectoral engagement to identify livelihood solutions for GBV survivors, despite limited employment options [41]. In Tanzania, GBV coordinators had contextually adapted risk mitigation strategies, resulting in GBV being well integrated in WASH and shelter sectors [46]. In several settings, involvement of GBV coordinators in assessments by shelter and WASH sectors, led to GBV risk mitigation measures such as locks and more secure tents [19, 41, 46]. UNFPA supported GBV service providers to integrate cash assistance as part of case management for Syrian refugee GBV survivors, though this was inadequate to address increased economic vulnerabilities during the COVID-19 pandemic [15].

##### Low commitment and accountability on GBV risk mitigation

Studies noted low levels of commitment to GBV minimum standards, limited understanding of how to operationalise the 2015 IASC GBV Guidelines, and weak accountability mechanisms to donors, humanitarian leadership, and beneficiaries [58] as well as weak linkages between gender equality and GBV in humanitarian action [55]. Recurrent challenges to integrating risk mitigation included: lack of knowledge and understanding on GBV risk mitigation; lack of clarity on staff roles in risk mitigation and assumptions that this was GBV experts’ responsibility; insufficient training on responsibilities, tools, and resources to support implementation; cultural barriers and biases among humanitarian actors; and limited incentives to address GBV on top of existing workloads [19, 39, 48]. Additionally, overwhelmed GBV focal points lacked capacity to effectively lead inter-agency coordination, and simultaneously integrate GBV risk mitigation [48]. An inter-agency evaluation of IASC GBV guidelines implementation in Jordan, Lebanon, Turkey and Iraq found mixed understanding about GBV risk among relevant staff and although donors were aware of the GBV guidelines, they rarely used them [58]. In Central African Republic and South Sudan, a general failure among sectors to integrate essential GBV risk reduction into emergency programming, reflected non-compliance with the GBV guidelines and absence of political will to address GBV [43]. Despite good progress in Lebanon, weak accountability mechanisms and follow-up, meant trainings did not necessarily translate into action within sector responses [41]. Furthermore, engaging refugee women in the design, management, and leadership of GBV risk mitigation measures appeared limited across setting, and accountability to affected women and girls minimal [11, 48, 58]. Humanitarian sectors responding to the Syrian conflict in 2015, rarely included meaningful or consistent accountability to refugees [58]. In Kenya’s Kakuma camp, refugee women found humanitarian GBV responses unhelpful, and continued employing their own systems for addressing GBV in their community [11, 59].

##### Non-compliance to GBV guidelines exacerbates GBV risks

Non-compliance of response actors to their responsibilities within the GBV guidelines exacerbated GBV risks for women and girls. One study noted minimum GBV risk reduction was overlooked during the Sierra Leone Ebola crisis, with cases and suspected cases not separated by sex in facilities, and few treatment centres able to treat pregnant women, resulting in denial of care and some women giving birth and dying on the street [43]. Lack of safe congregation spaces, lighting, or locks on toilets, tents, and showers increased GBV risks in displacement settlements [43]. In South Sudan, sexual violence risk factors included inadequate lighting, non-lockable, non sex-segregated toilets and showers, tents that unzipped from outside, and overcrowding [45]. MISP studies too, found multisectoral failures in essential GBV risk mitigation reporting that women felt unsafe using toilets at night in camps in Jordan and Nepal [33, 34]. Evaluations in Tanzania, Bangladesh and Brazil noted limited attention to ensuring safe access to shelter, firewood, and WASH facilities [19] and in South Sudan, women and girls were attacked and abducted while collecting firewood, water, and food [43]. In both Kenya and Haiti, lack of basic supplies and income increased vulnerability to transactional sex [45, 52].

#### Localisation

Fourteen sources explored issues of GBV localisation and inclusion of local actors in GBV coordination [15, 19, 21, 38, 40, 41, 43, 46, 48, 50–53, 55]. Despite significant global policy commitments, in practice, GBV localisation has been minimal, with little international funding channelled to local organizations, and several barriers and enablers were identified for local actors engaging in UN-led GBV coordination mechanisms.

##### Minimal progress on funding allocation to support the localisation agenda

In 2019, findings showed minimal GBV localisation in three of four contexts studied (i.e. Iraq, Nigeria, South Sudan), with only Turkey reporting high perceived localisation, which was necessitated by the lack of access of international organisations in Syrian cross-border operations [50]. Women-led local organizations were rarely allocated sufficient funding despite being recognised in policy commitments as key partners in GBV prevention and response, and little funding had been channelled to local organizations in general (e.g. just 0.4% of global GBV humanitarian assistance in 2015 and 0.3% in 2016) [43, 50, 55]. Without long-term, flexible, multi-year funding, local GBV organizations struggled to obtain independent funding, upgrade their internal management systems, or build reporting and accountability mechanisms, perpetuating the cycle [15, 51]. One study highlighted the lack of global good practice standards to guide localization efforts, which weakened implementation [50].

##### Barriers and enablers for engagement of local actors in UN-led GBV coordination mechanisms

Several sources noted cultural, linguistic and logistical barriers for local and national NGOs engaging in humanitarian coordination mechanisms, including meetings not being held in appropriate languages to facilitate participation [40, 46, 52]. In Haiti, for example, GBV coordination was criticised for holding meetings in English or French rather than Kreyol, thus excluding grassroots NGOs [52]. Exploitative partnerships included practices such as staff poaching and unequal pay for local actors versus UN or INGO staff, weakening technical expertise of local actors [50]. Challenges cited by humanitarian actors in working with local women’s rights organisations included their insufficient existing funding, capacity and ability to show impact, but also ideological concerns that such organisations were inherently ‘political’ and therefore inappropriate for engagement in impartial humanitarian GBV projects [51]. Several sources also cited patriarchal biases among international and national humanitarian actors as a major barrier to humanitarian actors working in partnership with women’s organisations [21, 50].

Nevertheless, some positive examples of engagement were highlighted. For example, in Lebanon, Jordan and South Sudan, UN organisations invested in capacity-building partnerships with local GBV actors, involving ongoing mentoring, which was seen as practical and sustainable [38, 41]. In Lebanon, local organisations expanded their geographical and services coverage rapidly as refugee numbers grew, by partnering with government, UN, and INGOs [53]. Using a system-building approach to implement long-term capacity building plans that strengthened government and civil society, UNICEF helped to create sustainable country-based GBV technical capacity in Lebanon and Jordan [38].

#### Data and information management

Eight sources noted data and information management as influencing effective coordination [15, 19, 38, 39, 41, 46–48]. Although requests for GBV prevalence data can delay funding and hamper progress, significant improvements have been noted since implementation of the GBVIMS, leading to enhanced coordination, funding allocation, service provision, and advocacy in many settings.

##### Requests for prevalence data

GBV coordinators are frequently asked for 'evidence' of GBV in the early days of a crisis, particularly in funding discussions. A 2016 multi-country study in Ethiopia, Tanzania and Lebanon, reported that donors and humanitarian leaderships’ need for ‘evidence’ of GBV prevalence was a fundamental challenge and when donors failed to earmark GBV funding at the beginning of a crisis, frontline implementation was delayed [46].

##### Adoption of gender-based violence information management system

A 2014 global evaluation noted that implementation of the GBVIMS had contributed to effective and safe collection, storage, analysis and ethical sharing of GBV data at country level [47]. Service providers were analysing and using GBV data for donor reports and fundraising, to identify gaps, better target and improve programmes, and enhance GBV coordination [47]. For example, analysis of time and location of GBV incidents in all camps in Dadaab refugee camp was used to enhance camp safety and reduce GBV risks [47]. GBVIMS was successfully implemented in Lebanon, Jordan and Iraq, allowing partners to track trends and target interventions, e.g. for child marriage in Lebanon [15]. UNFPA or UNHCR-hosted GBVIMS coordinators in Lebanon, Iraq, and Jordan, provided regular trend analyses that enhanced advocacy, coordination, and service provision [15]. Effective country-level rollout of GBVIMS was facilitated by strong technical support, country level ownership, a phased approach, strong and dedicated coordinators and existing interagency coordination [47]. Nevertheless, data management challenges were noted across settings, including varied reporting capacity, accuracy of data, and sharing restrictions that impacted quality and effectiveness [15, 19, 38]. Poor data management, in Uganda in 2015 and Tanzania in 2018, meant that data could not be used for planning [19] and national partners in Central African Republic used GBVIMS but lacked data collection expertise [38]. Engagement of national government by UN agencies was recommended to sustain GBVIMS, particularly as countries transitioned to recovery phases [47].

#### Coordination to support service delivery

Twenty-six sources included reflections on the importance of GBV coordination to support service provision [11, 15, 19, 21, 32–34, 36–48, 50, 52–55, 59]. Although specialist GBV services remain insufficient across emergency settings, emergencies can present opportunities for expanding and contextually-adapting services.

##### Insufficient specialist GBV services and trained staff

Many specialist GBV services, such as CMR and MHPSS, were insufficient across settings and lack of trained staff hampered services delivery in many contexts. In Central African Republic, Uganda, South Sudan, Iraq, and Sierra Leone, establishment of essential GBV services was hindered by insufficient availability prior to the crisis, slow deployment of GBV experts, limited funding, and sometimes weak advocacy for GBV prioritisation [38, 43, 54]. In Pakistan, humanitarian actors did not prioritise GBV services during 2010 floods, deeming them inappropriate given strict traditional gender norms [42]. During Sierra Leone’s Ebola epidemic, GBV services, provided through the public health system, were severely disrupted, and although GBV cases increased, specialist services remained inadequate [43]. CMR was only partially available during Nepal’s earthquake response, with gaps in availability of HIV prophylaxis and qualified staff [34] and in Jordan, access to CMR for Syrian refugees was limited by lack of emergency contraception, HIV prophylaxis, trained staff and a national protocol [32, 33]. In Ethiopia and Bangladesh, specialist GBV services were sometimes available for refugees but inadequate for IDPs and host communities [19, 46]. Specialist GBV services in Tanzania were short-term without sufficient follow up and limited shelters for GBV survivors in Lebanon, resulted in women returning to abusive partners [41, 46]. Lack of trained and qualified staff to deliver GBV services and limited female staff to treat women according to their cultural beliefs was an issue in several settings [32, 45, 54]. In Northern Syria, security challenges prevented doctors from crossing into Turkey for training and trainers from entering Syria, resulting in a lack of adequately trained medical providers for GBV survivors [58]. Importantly, lack of access to legal services prevented survivors from disclosing GBV incidents and in several settings, adolescent girls were at heightened risk for many forms of GBV, but rarely received tailored GBV interventions [38, 50, 54].

##### Emergencies present opportunities for expanding and contextually adapting GBV services

In Central African Republic, Jordan, Lebanon, Uganda, Somalia, and South Sudan, humanitarian GBV responses expanded GBV services provision and access, especially CMR, MHPSS, safe spaces, and community outreach [38, 41, 53, 54]. In Northern Uganda, the GBV sub-cluster enhanced GBV services quality through common approaches to provider training, monitoring, and standards and enhanced GBV information and services resulted in increased numbers of survivors seeking care [39, 40, 54]. GBVIMS in several settings helped to identify gaps in service provision and advocate for services [47]. Structured volunteer networks developed from refugee and host communities, in some settings, helped to improve community knowledge of GBV, services availability, referrals, and to monitor trends and an urban refugee women’s network in Turkey helped to strengthen confidence among refugees and to improve understanding of their rights [19]. In addition, UNHCR adapted services in Lebanon to reach dispersed urban populations through mobile outreach volunteers and innovative communication strategies [19, 41]. To improve participation and reduce stigma related to accessing GBV services in Somalia, South Sudan, Lebanon, Jordan, and Nepal, GBV-related activities were implemented in women and girls safe spaces, defined as a space which ensures the physical and emotional safety of women and girls [38, 60]. CMR training in Lebanon, was conducted with all staff at health facilities, not just medical staff, to ensure that survivors were uniformly treated in a survivor-centred manner [38]. In Georgia, UNHCR culturally adapted psychosocial services through group activities, building support networks, promoting skills building and economic empowerment [48]. Training, mentoring and support was provided to local NGOs in South Sudan, without previous GBV experience, to increase the number of organisations qualified to provide MHPSS, awareness raising, and referral of survivors [38]. UNHCR improved coordination with government and NGOs in Tanzania, to establish more effective legal services for survivors [48]. In the Syrian response, GBV interventions were adapted to the COVID-19 pandemic to maintain access to services through mobile and online modalities [15].

##### GBV prevention for long-term impact

GBV prevention programming is essential for long-term impact but often deprioritised in emergencies. Long-term GBV reduction requires addressing root causes, namely gender inequality and unequal power relations, and is often seen as too complex and long-term to implement in emergency contexts [21, 50]. In several settings, GBV response dominated GBV prevention because humanitarian agencies prioritised life-saving services, highlighting the need for increased investment in GBV prevention to address the root causes of GBV [15, 19, 39]. UNHCR community-based prevention activities showed promise but were small scale [19]. For example, 84% of women and adolescent girls participating in empowerment activities in Lebanon reported a greater sense of empowerment [19, 41]. More agencies reported exploring prevention or gender equality issues in protracted crisis contexts. For example within the protracted Syria response a progressive shift from service delivery, to risk mitigation, to prevention initiatives challenging harmful social norms, was enabled by multi-year, predictable funding [15]. Robbers et al. noted that the active involvement of female refugees in the design, planning and implementation of sexual violence preventative measures, increased empowerment and ownership of programmes and helped to transform harmful gender norms [59]. In 2019, the Women’s refugee council raised concerns about the increasing separation of work on GBV and gender equality in the humanitarian system, representing a missed opportunity for GBV prevention [55].

## Discussion

To our knowledge, this review is the first to explore GBV coordination in emergencies and revealed the near absence of academic literature systematically examining the effectiveness of GBV coordination. However, by maintaining a broad inclusion criteria and analysis framework, we were able to synthesise relevant findings for policy, practice, and research. Included sources spanned 2008 to 2020, and while the global policy context has evolved significantly in this period with many notable advancements, our findings highlight several remaining barriers to effective coordination, some of which were also noted in a 2021 gap analysis on GBV in humanitarian settings [61]. This review makes several important contributions including (1) a graphic overview of GBV coordination from global to frontline levels; (2) an evidence-informed framework on facilitators and barriers to effective GBV coordination; and (3) recommendations for strengthening GBV coordination in emergencies and for further research on this important topic (Table 4).

**Table 4:** Recommendations to enhance effectiveness of GBV coordination in diverse emergency settings

Dimension of GBv coordination framework	Recommendations	Target groups
Implementing a GBV sub-cluster	1. Ensure funding of dedicated long-term GBV positions at frontline, national, and global levels, including during public health emergencies	Donors, international & national GBV actors
	2. Adapt guidance and tools developed by GBV AoR for application in refugee and public health emergencies	GBV AoR, UNHCR and WHO
	3. Improve inter-sectorial engagement by deploying interagency coordinators early	Donors, international & national GBV actors
	4. Adapt coordination efforts to context to improve both effectiveness and sustainability	GBV AoR and UNHCR
	5. Research GBV coordination in diverse humanitarian and public health emergencies to provide more robust evidence on what influences effective GBV coordination in diverse settings	Researchers and donors
	6. Conduct research to understand strong leadership and effective coordination in the context of GBV	GBV AoR and Researchers
Prioritisation, advocacy and access to resources	7. Increase multi-year and flexible funding, especially in protracted emergencies	Donors and International GBV actors
	8. Proactively address patriarchy, and power imbalances which limit GBV prioritization and involvement of women-led organization's in coordination	Donors, international & national humanitarian actors
Risk mitigation and integration	9. Improve integration of risk mitigation across sectors through dedicated GBV specialists focused on supporting multi-sectorial integration and accountability	Donors, international & national GBV actors
	10. Improve engagement with beneficiaries to identify GBV risks, adapt services and promote bidirectional communication and accountability on mitigating risks	Donors, international & national GBV actors
	11. Mandate that GBV risk mitigation activities be included and budgeted in all funding proposals, with monitoring and evaluation	Donors, international & national GBV actors
	12. Train public health responders on GBV risk mitigation	WHO and GBV AoR
Localization	13. Strengthen subnational coordination mechanisms that engage and facilitate the leadership of local actors	Donors, international & national GBV actors
	14. Invest in partnerships to build both GBV technical capacity of frontline actors and to strengthen management systems to be eligible to receive international funding	Donors, UN & international GBV actors
	15. Increase funding allocations to national and local organisations	Donors, UN & international GBV actors
Data and information management	16. Limit requests for GBV prevalence data which delay funding allocation hampering GBV responses	Donors and humanitarian leadership
	17. Continue to improve the GBVIMS platforms and translate innovations across contexts	GBV AoR and Researchers
Coordination to support service delivery	18. Strengthen evidence on how GBV coordination addresses the needs of marginalised groups (eg, adolescent girls, boys, LGBTIQ +)	GBV AoR and Researchers
	19. Increase investment in context appropriate GBV prevention programming, especially in protracted emergencies, through multiyear planning and funding	Donors, GBV AoR and UNHCR
	20. Develop practical guidance on approaching culturally sensitive issues such as shame, stigma and social norms within GBV programming, including on training health care workers	GBV AoR

The overview of GBV coordination graphic highlights the complex network of organisations and actors involved in addressing GBV in emergencies. The UNFPA-led GBV AoR take the lead in non-refugee settings and have developed comprehensive guidance, standards and toolkits for application in the cluster system, in addition to providing training and technical support [8]. Coordination in refugee settings, however, is led by UNHCR, and it is not clear from the available literature, if GBV AoR guidance and tools are applied systematically in refugee settings or if technical support is provided. In addition, much guidance relates to traditional camp settings, but increasingly refugees and IDPs live in urban and peri-urban contexts, creating additional context-specific GBV risk and access challenges that deserve attention [62]. Furthermore, our review demonstrates a gap in awareness to GBV coordination in public health emergencies, when coordination is under the World Health Organization (WHO). Although it is widely accepted that risk factors for GBV are magnified during infectious disease outbreaks [14], only three sources presented reflections on GBV coordination in outbreaks. Further research is needed to learn from and adapt innovative GBV coordination mechanisms and service provision approaches implemented during the COVID-19 pandemic.

Findings indicated major improvements in GBV coordination in emergencies, attributed to rapid activation of coordination mechanisms and organisational investments in building and deploying GBV coordination experts [63]. Deploying GBV specialists early, strengthened coordination, donor confidence, GBV prioritisation and funding allocation across settings. Thus, ensuring funding for dedicated, experienced, long-term GBV coordinators should be promoted in all kinds of emergencies, including public health. WHO is augmenting efforts to address GBV in health emergencies, including through the deployment of GBV advisors at regional, global and country-level and to newly graded health emergencies, which deserves further investment and expansion [64, 65]. While the concepts of strong coordinators and good leadership qualities appeared to be important for effective GBV coordination, more research is needed to understand and characterise these terms in the context of GBV.

Major gaps remain between global GBV policy commitments and funding allocations, with a lack of prioritisation, commitment and accountability across the humanitarian sector. GBV is consistently de-prioritised, with less than 1% of humanitarian funding allocated to the GBV sector over the past 5 years [61]. Our findings emphasised the importance of adequate GBV funding and human resources, alongside multi-year, flexible funding for protracted emergencies [4, 57, 61]. Still, existing humanitarian financing systems are unaligned with the needs, with short-term, inflexible funding, limiting deployment of long-term, senior GBV coordinators, inclusion of local actors, and investment in GBV risk mitigation and prevention. In the context of the COVID-19 pandemic, increased multi-year and flexible funding is critical to meet increasing and emerging GBV needs [15].

Significant benefits of investing in subnational coordination include faster and more contextually-relevant decision-making and greater participation by local actors—particularly civil society—to advance the localisation agenda, but requires strengthening in emergencies [4, 49, 66–69]. Despite global commitments to GBV localisation, progress has been slow and uneven, with little evidence suggesting local actors have been meaningfully included in GBV coordination efforts or received adequate funding [21, 50]. Local actors have greater understanding of context, are embedded in the affected populations, and with language and cultural knowledge, can navigate complex socio-political dynamics more easily, yet global targets to increase local organisations’ funding, from under 3 to 25% by 2020, have not been achieved [21, 50, 57]. Security, movement restrictions and access concerns in many emergencies, including COVID-19 restrictions, underscore the need for investment in local GBV technical capacity-building [21, 70–72]. Good examples from Syrian refugee responses in Lebanon, Jordan, and Turkey could be used as case studies. Inclusion of women-led organizations, and women from affected communities, is similarly crucial, to address GBV prevention and risk mitigation in culturally appropriate ways [21, 61]. Challenges of funding access, inequitable power dynamics and patriarchal attitudes within the humanitarian sector require targeted attention at global and country levels [61].

Limited commitment to GBV risk mitigation across sectors suggested stronger inter-sectorial engagement and improved inter-agency accountability systems are needed to improve multi-sectoral resourcing and attention [4, 40, 49]. The humanitarian system has made some progress integrating GBV risk mitigation since 2016, with the roll-out of the revised IASC GBV Guidelines in multiple countries, and initiatives such as the Real-Time Accountability Partnership [73]. Still, risk mitigation activities are often seen as under the remit of the GBV sector, rather than integrated across all sectors [61]. As non-GBV specialists may not have the required expertise to mitigate GBV risks [61], deploying GBV risk mitigation specialists with dedicated time and funding could help sectors to meet their responsibilities using a mentorship approach. Donors too can improve systems by requiring that GBV risk mitigation activities be included and budgeted in all funding proposals, with monitoring, evaluation, and follow-up on reporting. In addition, GBV guidelines are not systematically integrated in public health emergencies and more efforts are needed to ensure that public health responders understand and address their responsibilities. Furthermore, inclusion of, and accountability to, affected populations in development and monitoring of risk mitigation measures requires investment [61].

Collecting and sharing GBV information is both extremely challenging and important in emergencies. Despite global guidance, our review highlighted that donor requests for ‘evidence’ of GBV remains a consistent challenge, delaying funding allocation and GBV responses [7, 46]. Furthermore, unethical practices such as donors requiring access to individual survivor information can put survivors at increased risk [61]. The implementation of GBVIMS since 2008 has provided notable improvements, with innovative digital platforms rolled out across multiple contexts, which could be duplicated elsewhere. Importantly for GBV coordination, GBVIMS helps to inform programmatic decision-making for service providers and inter-agency working groups, improve donor reporting and fundraising, and strengthen advocacy efforts [47].

Effective GBV coordination ensures comprehensive multi-sectorial, survivor-centred services, strong referral mechanisms, and collaborative, culturally-appropriate programming. However, our review highlighted significant gaps in both availability of services and access of survivors across emergency settings. Stigma, shame and lack of appropriately trained staff are common barriers to survivors accessing GBV services, and practical guidance on approaching these culturally-sensitive issues within GBV coordination structures is needed [19, 48]. While there have been considerable efforts to improve coordination and programming strategies between the GBV and child protection sub-sectors, including the Child and Adolescent Survivors Initiative, adolescent girls are still often overlooked in GBV programming [61]. In addition, our review highlights a lack of evidence on how the coordination system accounts for the needs of specific groups such as people with disabilities, LGBTIQ + and marginalised populations, including migrants and sex workers. While GBV prevention is essential for long-term impact, it is rarely prioritised in emergency responses, but multi-year, predictable funding, especially in protracted crisis, can encourage investment in culturally-appropriate prevention programming [61, 74]. Linkages between gender equality and GBV require strengthening and investments are required in translating the increasing empirical evidence about ‘what works’ to prevent GBV in humanitarian settings [55, 61].

In settings without existing GBV coordination mechanisms, emergencies can provide an opportunity to introduce GBV coordination and expand services [7]. Particularly in protracted emergencies where humanitarian actors are required to support both immediate and longer-term needs, GBV coordination and services have been embedded and expanded in several settings. Nevertheless, advances in GBV coordination are not routinely sustained and built upon, with GBV coordination and service delivery often dependent on international funding and leadership, coupled with weak government commitments to institutionalising services and systems [38]. In settings with pre-existing GBV coordination structures, merging emergency GBV coordination into government and civil-society structures is recommended, however, in reality, implementation is often challenging [7, 8, 66, 67, 75, 76]. Finally, GBV coordination efforts should be contextually nuanced and build on existing government, and civil society networks to improve both effectiveness and sustainability [66, 77].

### Limitations

This study has several limitations and should be interpreted accordingly. Firstly, we only included sources within our search and language capacity, and it is possible that other relevant sources were inaccessible due to search terms or unavailable electronically. Secondly, only the first author searched and selected sources, however, discussion and oversight of other authors minimised bias. Finally, sources were not excluded on evidence quality, allowing inclusion of a broader range of data from peer-reviewed and grey literature.

## Conclusions

While GBV coordination is increasingly recognised as vital to global efforts to respond to, mitigate and prevent GBV, it is rarely researched, demonstrated by the lack of peer-reviewed sources, with literature on GBV coordination during public health emergencies particularly scant. Recommendations to strengthen GBV coordination include to, increase multi-year and flexible funding for GBV across emergencies, fund dedicated GBV coordination positions in all emergencies, build the global GBV coordination workforce including for deployment in public health emergencies, strengthen subnational coordination mechanisms, expand inclusion and leadership of national and local actors and channel more funding to these organisations. In addition, guidance and tools developed by the GBV AoR should be adapted for application in refugee settings and public health emergencies, and investment in context appropriate GBV risk mitigation and prevention should be promoted through multiyear planning and funding, especially in protracted emergencies. We present a series of recommendations (Table 4) to improve effectiveness of GBV coordination across emergency settings. The evidence-based framework for effective GBV coordination presented above, can help guide further research to explore effective GBV coordination in diverse emergencies.

## Supplementary Information

**Additional file 1.** **Box 1**: Search strategy.

## Data Availability

All data generated or analysed during this study are included in this published article [and its additional files].
