# Supplementary material for: Gender-based violence (GBV) coordination in humanitarian and public health emergencies: a scoping review
Source: Confl Health. 2022 Jun 28;16:37. doi: 10.1186/s13031-022-00471-z (PMC9238064; doi:10.1186/s13031-022-00471-z)
Supplement: Supplementary file 1 — Additional file 1. Box 1: Search strategy. [file 13031_2022_471_MOESM1_ESM.docx]

**Additional File 1**

## **Box 1. Search strategy**

| 1. **Humanitarian/Emergency response**   Humanitarian system OR Humanitarian response OR Emergency response OR Crisis response OR emergenc* OR natural disaster* OR refugee* OR conflict setting* OR displacement OR Internally displaced OR internally displaced population*   1. **Coordination in Humanitarian/Emergency response**   coordinat* OR co-ordinat* OR cooperat* OR co-operate* OR collaborat* OR coordination OR collaboration OR cooperation OR “humanitarian coordination” OR “coordination mechanism” OR “cluster system” OR “cluster approach”   1. **Gender-based Violence (GBV)**   GBV OR gender-based violence OR violence against girls OR violence against women OR “violence against women and girls” OR VAWG OR domestic violence OR intimate partner violence OR IPV OR partner violence OR abuse of women OR hono?r killing OR rape OR sexual violence OR child marriage OR forced marriage OR early marriage OR sexual trafficking OR “sexual exploitation and abuse” |
| --- |
